# Mass spectrometry-based proteomic approaches to study pathogenic bacteria-host interactions

**DOI:** 10.1007/s13238-015-0136-6

**Published:** 2015-02-27

**Authors:** Yufei Yang, Mo Hu, Kaiwen Yu, Xiangmei Zeng, Xiaoyun Liu

**Affiliations:** Institute of Analytical Chemistry and Synthetic and Functional Biomolecules Center, College of Chemistry and Molecular Engineering, Peking University, Beijing, 100871 China

**Keywords:** mass spectrometry, proteomics, bacterial infection, host-pathogen interactions

## Abstract

Elucidation of molecular mechanisms underlying host-pathogen interactions is important for control and treatment of infectious diseases worldwide. Within the last decade, mass spectrometry (MS)-based proteomics has become a powerful and effective approach to better understand complex and dynamic host-pathogen interactions at the protein level. Herein we will review the recent progress in proteomic analyses towards bacterial infection of their mammalian host with a particular focus on enteric pathogens. Large-scale studies of dynamic proteomic alterations during infection will be discussed from the perspective of both pathogenic bacteria and host cells.

## Introduction

Infectious diseases are caused by pathogenic microorganisms such as viruses, bacteria, or fungi, and represent major health risks for humans as well as animals and plants (Khabbaz et al., 2014). Despite intense efforts to develop novel strategies to combat and prevent infections, newly emerging infectious diseases as well as recurrence of previously controlled ones (i.e., due to increasing antibiotic resistance) continue to pose a great challenge for our fight against pathogens (Khabbaz et al., 2014). Central to infectious disease research, better understanding of the functional interface between pathogenic microbes and their host cells is strongly desired. In fact, exact molecular mechanisms governing different stages of infection (i.e., adhesion, invasion, replication, etc.) are still poorly understood (Cossart and Sansonetti, 2004). This lack of knowledge in understanding disease pathogenesis impedes the development of new diagnostic and therapeutic strategies.

The intricate interplay between host and pathogens involves hundreds to thousands of proteins from both sides (Hartlova et al., 2011; Zhang et al., 2005). Over the years, most of research efforts have focused on characterization of individual bacterial virulence factors and their interacting host targets by traditional genetic and biochemical methods. These classic approaches have contributed tremendously to our understanding of many important aspects of infection biology. Nevertheless, such studies alone cannot explain the complex multifactorial nature of host-pathogen interactions (Bumann, 2009). Rather, systems-level analyses enable a panoramic view of the functional host-pathogen interplay, complementing significantly the traditional reductionism-dominant research (Walduck et al., 2004). Transcriptomic studies have been performed for many years and yet direct measurements on the final gene products, proteins, are highly desirable because of poor correlation between mRNA and protein levels due to extensive post-transcriptional regulations.

**Figure 1:**
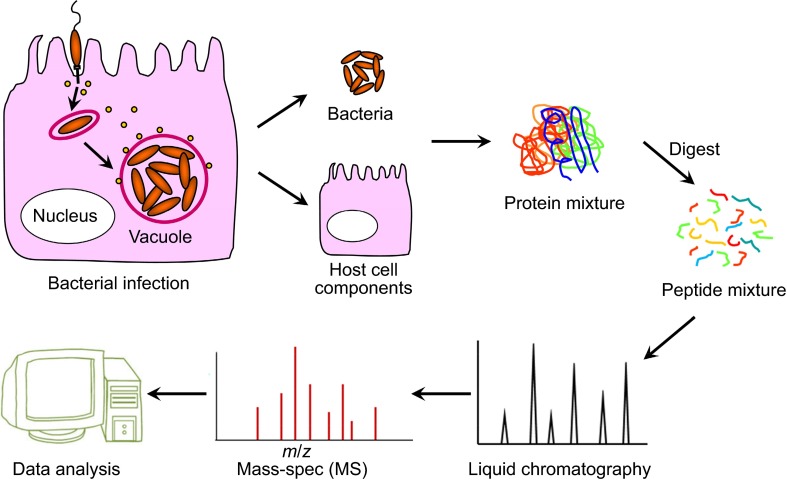
**A general workflow of proteomic studies of host-pathogen interactions**. Upon infection of host cells (with intracellular bacteria as an example), bacterial pathogens were physically isolated from host cells and subjected to high-throughput proteomic analyses by LC-MS-based approaches. Host cellular components can also be analyzed, though extensive sample fractionations were necessary for sufficient proteome coverage (i.e., subcellular enrichment and protein gel separation). Protein samples are enzymatically digested into peptide mixtures prior to LC-MS measurements for both qualitative and quantitative analyses

Over the years, two-dimensional (2-D) gel electrophoresis followed by MS identifications of individual protein spots has been utilized for the prototype proteomic studies (Curreem et al., 2012; Rabilloud et al., 2010). In addition to being time-consuming, however, 2-D gel can never meet the challenges of complex biological samples due to its rather limited sensitivity and dynamic range (Jafari et al., 2012). Fortunately within the last decade, MS-based proteomics has evolved into a high-sensitivity high-throughput approach for quantitative examination of proteins from any biological system on a global scale (Fig. 1). The core platform of this technology is liquid chromatography coupled with tandem mass spectrometry (LC-MS/MS), in which complex protein samples are enzymatically digested into peptides prior to chromatographic separation and MS identifications (Cravatt et al., 2007). Importantly, the field of MS-based proteomics has witnessed major technical breakthroughs such as the advent of high-resolution Orbitrap mass spectrometers. Currently, the state-of-the-art proteomic technologies are capable of measuring several thousands of proteins within a few hours, which permits us to significantly increase the analytical depth as well as coverage in complex proteome analyses (Ding et al., 2013; Geiger et al., 2012; Weekes et al., 2014).

In fact, an increasing number of studies have used LC-MS/MS based proteomic approaches to investigate the consequences of infection for both pathogens and their host (Fig. 1). This review will focus on the latest progress made in this area towards systems-level characterization of pathogen and host proteomes in particular during enteric bacterial infection (Table 1). Moreover, we will discuss the challenges/limitations and future directions of MS-based proteomics in understanding host-pathogen interactions on the molecular level.

**Table 1:** Example studies of host-bacteria interactions using MS-based proteomics

		Work description	References
Bacterial pathogens	Grown in bacteriological media	Compared the proteome of *S*. Typhimurium in different growth phases and culture media	Adkins et al. 2006
		Compared the proteome of *S.* Typhi in different growth conditions	Ansong et al. 2008
		Contrasted *S *. Typhimurium protein expression in two *in vitro* conditions	Shi et al. 2009a
	Upon interaction w/host	Surveyed *S.* Typhimurium proteome in mouse infection models of typhoid fever and enteritis	Becker et al. 2006
		Time-course studies of *S.* Typhimurium protein expression in infected macrophages	Shi et al. 2006
		Proteomic analyses of *Campylobacter jejuni* in infected host epithelial cells	Liu et al. 2012
		Analyses of *Shigella flexneri* proteome in infected Henle cells	Pieper et al. 2013
Host cells	Expression profiling	Proteomic response of macrophages upon *S.* Typhimurium infection	Shi et al. 2009b
		Proteomic studies of intestinal epithelial cells infected by enteropathogenic *E. coli*	Hardwidge et al. 2004
		Proteome analysis of purified *Legionella*-containing vacuoles from host cells	Urwyler et al. 2009 Hoffmann et al. 2014
	PTMs	Phosphoproteomics of *S.* Typhimurium infected host cells	Rogers et al. 2011
		Phosphoproteomics of host cells upon infection by SPI2-deficient *S.* Typhimurium	Imami et al. 2013
		Profiling of the host phosphoproteome upon *Shigella flexneri* infection	Schmutz et al. 2013
		Covalent modifications of a host small GTPase Rab1 mediated by *Legionella pneumophila* effector proteins	Muller et al. 2010 Mukherjee et al. 2011

## Proteomic analyses of bacterial pathogens

As one of the primary species of pathogenic microorganisms, bacterial pathogens cause many life-threatening infectious diseases associated with human beings (Cossart and Sansonetti, 2004). The accomplishment of genome sequencing of various microorganisms has laid the foundation of proteomic studies (Gygi et al., 1999). Compared to eukaryotic cells, the proteome of bacteria is significantly more approachable due to their relatively compact genome. Furthermore, unlike eukaryotes where alternative splicing and post-translational modifications (PTMs) are common occurrence, the complexity of bacterial proteome can be considered as comparable to that of their genome. Therefore, most proteomic work in host-pathogen interactions was carried out on bacterial pathogens, which can be further divided into two mainstreams: (1) bacteria grown in bacteriological media (i.e., free-living); (2) bacteria upon interactions with host cells (i.e., intracellular or *in vivo* during infection) (Cash, 2011).

## The proteome of bacteria grown in bacteriological media

Because MS measurements of bacterial proteins against overwhelming background of host proteins are still technically challenging (Schmidt and Volker, 2011), vast majority of work thus far falls into examination of the bacterial proteome in bacteriological media. A typical experimental design is to alter growth conditions to somewhat mimic the context of host cellular environment, and then examine differential protein expression of pathogenic bacteria due to such perturbations. For example, numerous studies were carried out to characterize bacterial pathogens that were exposed to a wide variety of environmental challenges, such as fluctuations in temperature (Zhu et al., 2010), pH (Stancik et al., 2002; Yohannes et al., 2004), osmotic pressure (Weber et al., 2006), oxidative stress (Kim et al., 2010), and nutrient limitations (Albrethsen et al., 2013). By analyzing differences in proteomes, one could learn about bacterial adaptation mechanisms in response to a given environmental perturbation.

*Salmonella enterica* (*S. enterica*) is frequently used as a model bacterial pathogen and therefore its proteome has already been intensively studied. *S. enterica* serovar Typhimurium (*S.* Typhimurium) is a common cause of gastroenteritis and *S.* Typhi infection in humans leads to typhoid fever and considerable morbidity as well as mortality (Haraga et al., 2008; Ohl and Miller, 2001). Most work on *Salmonella* proteome was carried out by Smith, Heffron and their co-workers (Adkins et al., 2006; Ansong et al., 2008; Ansong et al., 2009; Brown et al., 2012; Shi et al., 2009a). In 2006, they reported the global analyses of *S.* Typhimurium protein expression from distinct strains (LT2 and ATCC 14028) cultivated in different growth conditions (Adkins et al., 2006). They compared *Salmonella* proteomes in different growth phases (logarithmic and stationary), and an acidic, magnesium-depleted minimal medium (MgM) (to mimic the conditions within the *Salmonella*-containing vacuoles in infected macrophages). These three conditions in principle permit the comparison of standard laboratory cultures to conditions that favor the expression of *Salmonella* virulence factors. In total, 2343 bacterial proteins from the LT2 strain were detected, with 1589 and 1995 proteins present in all three growth conditions and at least two conditions respectively. They focused their attention on bacterial products that were detected exclusively in a single condition. In particular, they found that a unique set of proteins were induced under the MgM growth condition, including Mg^2+^ transporters and propanediol metabolic proteins (Pdu proteins). Furthermore, a protein from *Salmonella* two-component system, PhoR, was also found to be highly induced. They further extended this comparative study to another more virulent strain ATCC 14028, and found strikingly that the abundance of Pdu proteins was even higher than that of the LT2 strain, thereby suggesting a possible link of *pdu* genes with *S.* Typhimurium pathogenesis. The proteins encoded by the *pdu* operon confer *Salmonella* the ability to grow with propanediol as a sole carbon source. However, the authors also noted that propanediol might not be readily available in macrophages, as it is one of the breakdown products of rhamnose and fucose. Therefore, further studies would be needed to precisely determine the contribution of the *pdu* operon to *S.* Typhimurium pathogenesis. Nevertheless, differential proteomic analyses of bacterial strains cultured under distinct growth conditions open a new window for us to study the mechanisms of pathogens’ adaptations to specific environment on a systems-level.

In the following years, the same team analyzed the proteome of *S.* Typhi (a different serotype) in the three growth conditions described above (Ansong et al., 2008). They found a group of 50 proteins (such as CdtB and HlyE) were exclusively expressed in *S.* Typhi Ty2, especially under the MgM growth condition. These proteins may play important roles in *S.* Typhi pathogenesis and its human host restriction. A year later, they surveyed the *S.* Typhimurium proteome again under two more growth conditions (termed MgM Shock and MgM Dilution) (Shi et al., 2009a). They demonstrated that MgM Shock up-regulated proteins that are usually induced at low oxygen levels, while MgM Dilution induced the expression of virulence factors and proteins that are associated with biotin and thiamine biosynthesis. Furthermore, they examined protein expression of *Salmonella* *hfq* and *smpB* deletion mutants (Ansong et al., 2009), and also *Salmonella* subcellular proteome (Brown et al., 2012). At the same time, many other groups also contributed to studies of *Salmonella* proteome in bacteriological media (Di Pasqua et al., 2010; Kim et al., 2010; Sonck et al., 2009; Yu and Guo, 2011).

## Bacterial proteome upon interactions with host cells

Though proteomic studies of bacteria grown in bacteriological media have been routinely practiced for years, analyses of their protein expression *in vivo* or intracellularly within infected host cells are still rare (Schmidt and Volker, 2011). Proteomic measurements of bacterial pathogens upon interactions with host cells are much more difficult, because limited amounts of bacterial proteins are present together with overwhelming amounts of host components (Schmidt and Volker, 2011; Sengupta and Alam, 2011). Therefore, a prerequisite of such studies is effective isolation of bacterial pathogens from host cells (Paape et al., 2008). As reviewed by Schmidt and Völker, currently three strategies have been developed including differential centrifugation (Fernandez-Arenas et al., 2007), immunomagnetic separation (Twine et al., 2006), and fluorescence-activated cell sorting (FACS) (Becker et al., 2006). Differential centrifugation has been most widely practiced, and yet further improvements of all these methods are highly desirable because contamination by host proteins is still a serious problem (Schmidt and Volker, 2011).

To our knowledge, till now only two studies reported *Salmonella* proteome upon interactions with the host. In 2006, Becker et al. successfully isolated GFP-expressing *S.* Typhimurium by FACS from mouse tissues, and identified 370 and 835 *Salmonella* proteins from models of typhoid fever and enteritis, respectively (Becker et al., 2006). Most observed proteins were metabolic enzymes, majority of which were found to be nonessential for *Salmonella* virulence, thereby reflecting extensive metabolic redundancies. The first quantitative study of *Salmonella* proteome during infection was performed by Smith and Heffron groups (Shi et al., 2006). *S.* Typhimurium was isolated from RAW264.7 macrophages by centrifugation, and protein expression at various time points post infection was analyzed. In total, they identified 315 *Salmonella* proteins and found 39 significantly altered proteins. Nevertheless, both studies suffered from rather limited proteome coverage given the fact that *Salmonella* genome encodes ~4500 proteins. In addition, a sizable fraction (up to 50%) of detected proteins was still derived from the host, which in turn contributed, at least in part, to the insufficient proteome coverage.

A few years ago, we sought to develop a generic, highly efficient approach for isolation of intracellular bacterial pathogens. We chose a cell lysis condition (0.1% Triton X-100) in which only the plasma membrane of host cells can be solubilized with minimal impact on bacteria and nuclei, and carefully optimized each centrifugation step. Additionally, we introduced a brief but harsh washing step in the end for pelleted bacteria, which was found to markedly reduce host contaminants. We applied this strategy to analyze *Campylobacter jejuni* within infected host epithelial cells (Liu et al., 2012). In total, we identified 1428 bacterial proteins corresponding to 86% of the entire *C. jejuni* proteome. More importantly, <15% of all detected proteins were found to be of host origin, thereby suggesting minimal host contamination. Interestingly, we found that vast majority of differentially regulated proteins at 20 h post infection were down-regulated, which strongly supports a notion that *C. jejuni* underwent a significant metabolic downshift within host cells. Our findings indeed correlated well with previous observations that *C. jejuni* enters a dormancy-like state upon internalization and doesn’t replicate within cultured epithelial cells. Moreover, our proteomic data also suggested that *C. jejuni* reprogrammed its anaerobic respiration pathways by favoring fumarate as a final electron acceptor. Further genetic disruption of fumarate respiration significantly reduced *C. jejuni* intracellular survival within infected host cells.

Recently, intracellular proteome of *Shigella flexneri* during infection has also been reported (Pieper et al., 2013). *Shigella* is a major cause of morbidity and mortality of dysentery worldwide (Kotloff et al., 1999). Intracellular *S. flexneri* strain 2457T was isolated from infected Henle cells and compared to extracellular populations. An average of 1170 *S. flexneri* proteins were detected per experiment, among which 190 proteins were significantly altered at 3 h after invasion, including those associated with invasion and cell-to-cell spread (i.e., Ipa, Mxi and Ics proteins). In addition, iron acquisition systems and Fe-S cluster assembly proteins were up-regulated as well, thus indicating iron starvation in the host. Importantly, marked alterations in metabolic pathways in response to the intracellular environment were also evident such as elevated levels of glycogen biosynthesis and mixed acid fermentation enzymes. Subsequent mutational studies confirmed mixed acid fermentation pathways are important for *S. flexneri* intracellular growth as well as cell-to-cell spread. Collectively, proteomic analyses of intracellular bacteria or those isolated *in vivo* from infected animals clearly demonstrated their utility in revealing pathogens’ adaptation mechanisms while residing in the host (Kuntumalla et al., 2011; Pieper et al., 2009; Suh et al., 2014).

## Proteomic analyses of host cells upon bacterial infection

During infection, while bacterial pathogens have to re-sculpt their own proteome in response to host environment, they also impact (induce or inhibit) the expression of certain host factors in a way that will benefit their own survival and replication. From another perspective, in order to combat bacterial infection host cells will actively reprogram their gene/protein expression (i.e., activation of innate immune systems) (Jenner and Young, 2005). Therefore, study of the dynamic host proteome upon infection allows us to understand either bacteria-targeted host cellular pathways or host-mounted defense strategies (to contain or eliminate bacterial infection).

In addition, host proteins are often subjected to post-translational modifications, which exert an additional layer of regulation. Indeed, bacterial pathogens are known to possess virulence factors that can mediate covalent modifications or directly modify host substrates (Salomon and Orth, 2013). For instance, many T3SS-delivered effector proteins harbor enzymatic domains that were found to be kinase/phosphatase and E3 ubiquitin ligase/deubiquitinase. Therefore, bacterial pathogens have evolved the capacities to directly hijack host phosphorylation or ubiquitination machineries in order to promote their survival in the host (Galan and Wolf-Watz, 2006).

## The dynamic expression of host proteome during infection

The host proteome is much more complex than that of bacterial pathogens because many more proteins are present with greater dynamic ranges. Thus far only a handful of studies reported host proteome upon infection. Rather, host transcriptome profiling has been more widely practiced in host-pathogen interactions (Jenner and Young, 2005). Nevertheless, proteins are the final gene products that carry out most of biological activities and hence proteomic studies are more informative in inferring relevant biology.

In 2009, Smith and Heffron groups carried out time course proteomic studies of RAW264.7 macrophages upon *S.* Typhimurium infection (Shi et al., 2009b). In total they detected 1006 macrophage proteins, and 244 proteins were significantly changed compared with non-infected controls. Those altered proteins were functionally diverse including antibacterial NO production, prostaglandin H_2_ synthesis, and regulation of intracellular traffic, thereby suggesting broad impact on host cells towards *Salmonella* infection. Similarly, Hardwidge et al. performed quantitative proteomic analysis of human Caco-2 intestinal epithelial cells upon infection with enteropathogenic *E. coli* (EPEC) (Hardwidge et al., 2004). EPEC is another enteric pathogen responsible for protracted and chronic diarrhea in children and much of its virulence is mediated through T3SS, which can inject effectors into host cells with the capacity to modulate actin dynamics as well as immune responses (Kaper et al., 2004). To study host responses induced by EPEC T3SS, they quantitatively profiled >2000 host proteins and found 264 proteins that showed marked differences in cells infected by WT and T3SS-deficient strains. Most of the differed proteins were involved in actin dynamics, cell adhesion, as well as G-protein signaling pathways. In particular, proteins associated with ion transport and ion channels were up-regulated during infection. They further confirmed some of the proteomic changes through Western blotting and immunofluorescence studies.

In addition to the whole proteome analysis, proteomic studies of host organelles (i.e., phagosome) have also gained attention because they can provide highly specific information about the dynamics of host compartments. Hilbi and co-workers described an elegant approach that allows isolation of highly purified *Legionella*-containing vacuoles (LCV) within *Dictyostelium discoideum* by combining magnetic immunoseparation and density gradient centrifugation (Urwyler et al., 2009). Follow-up LC-MS/MS analyses revealed many known LCV components as well as two novel proteins (Rab8 and Rab14) that are present on the vacuoles, indicating LCV also communicate with late secretory and endosomal pathways. Recently, the same group examined the LCV proteome in infected macrophages and found 13 Rab GTPases including six novel ones (Hoffmann et al., 2014). Knock-down of these Rab proteins by RNA interference was found to either restrict or promote intracellular bacterial growth. Additionally, they also identified many *Legionella* effectors and proteins that are associated with phosphorus metabolism. In principle, the approach described above can be readily applied to study other bacteria-containing phagosome and such studies are likely to shed new light on the complex biogenesis of bacterial vacuoles (Li, 2011).

## Global profiling of host protein modifications during infection

Post-translational modifications play an important role in the functional interplay between host and bacterial pathogens. As one of the most recognized modifications, phosphorylation is central to signal transduction in various biological processes. Successful pathogens are often capable of modulating host signaling pathways for their own benefit. From a technical perspective, phosphoproteomics is perhaps the most established area in large-scale MS profiling of PTMs (Thingholm et al., 2009). Because most modifications are present in low stoichiometry and would typically be discriminated by MS detection, a prerequisite of examining any modification on a global scale is the development of highly efficient enrichment strategies prior to MS measurements. Several classes of materials have been developed for specifically enriching phosphopeptides such as immobilized metal affinity chromatography (IMAC) and TiO_2_ (Thingholm et al., 2009).

Foster and co-workers applied a quantitative phosphoproteomic strategy to characterize bacteria-targeted host signaling pathways during *Salmonella* infection (Rogers et al., 2011). In total, 1973 phosphoproteins were detected corresponding to 9508 phosphorylation sites during the initial 20 min after *Salmonella* infection. Compared to the wild-type, infection with a *sopB*-deficient strain resulted in 35% decrease in the number of proteins with altered phosphorylation, thus highlighting the broad impact of a single T3SS effector on hundreds of host phosphorylation events. Last year, the same group reported the global impact of *Salmonella* Pathogenicity Island 2 (SPI2)-encoded effectors on the host phosphoproteome (Imami et al., 2013). When comparing WT and Δ*ssaR* (SPI2-deficient)-infected RAW264.7 macrophages, most altered phosphoproteins were involved in protein transport, regulation of actin and immune signaling. Interestingly, phosphoproteins that differed most in infected HeLa cells were associated with apoptosis and regulation of gene expression. Similar work on host phosphoproteomics was also carried during *S. flexneri* infection and thousands of phosphorylation sites were detected as well (Schmutz et al., 2013). Coincidentally, the authors also demonstrated that deletion of a single effector gene *ospF* affected phosphorylation status of over three hundred proteins.

## MS characterization of novel PTMS mediated by bacterial virulence factors

In addition to global profiling, MS has also been instrumental and indispensable in characterizing potentially novel modifications. Indeed, MS is one of the most reliable and efficient means of analyzing covalent modifications and importantly tandem MS (MS/MS) has been regarded as the gold standard for mapping modification sites. In bacterial pathogenesis, covalently modifying host proteins by bacterial virulence proteins has emerged as one of the most exciting areas in recent years (Salomon and Orth, 2013). For instance, the Shao group carried out a series of elegant studies demonstrating that bacterial pathogens are extremely versatile and ingenious in modulating host cellular pathways through covalent modifications. Newly identified host protein modifications by this group include irreversible removal of phosphate from phosphothreonine of mitogen-activated protein kinase (MAPK) by a *Shigella* effector OspF (Li et al., 2007), glutamine deamidation of NEDD8, cysteine methylation of ubiquitin-chain sensory proteins TAB2/3 and arginine GlcNAcylation of the death receptor complex by EPEC effectors Cif, NleE and NleB, respectively (Cui et al., 2010; Li et al., 2013; Zhang et al., 2011).

As a paradigm in this regard, covalent modifications of a host small GTPase Rab1 by *Legionella pneumophila* effectors have attracted considerable interest lately. *L. pneumophila* is a gram-negative facultative intracellular pathogen that can cause Legionnaire’s disease (Fields et al., 2002). Rab1 is a key regulator of membrane transport in the early secretory pathway of eukaryotic cells (Sherwood and Roy, 2013), and during infection it was targeted by multiple effector proteins such as DrrA/SidM and AnkX (Ingmundson et al., 2007; Machner and Isberg, 2007; Murata et al., 2006; Schoebel et al., 2009). Although initially identified as a guanine nucleotide exchange factor (GEF) of Rab1, strikingly DrrA was later found out to harbor a novel enzymatic activity by catalyzing the transfer of an AMP molecule to Rab1 (termed AMPylation) at least *in vitro* with purified proteins. Further tandem MS analysis of modified Rab1 decisively located the exact site of modification (Muller et al., 2010).

However, whether this novel Rab1 modification occurs during bacterial infection of host cells was still unclear. Most bacterial pathogens tend to deliver effector proteins at exceedingly low amounts to spatially highly-restricted locations, thereby rendering the pool of modified Rab1 extremely small. Considering Rab1 is not an abundant cellular component, MS measurements of its modifications within host cells can be technically more challenging. Nevertheless, Mukherjee and Liu et al. presented convincing MS evidence that DrrA indeed mediated Rab1 AMPylation during *L. pneumophila* infection (Mukherjee et al., 2011). Remarkably, they uncovered a potentially novel Rab1 modification, which was mediated by a different effector AnkX. Intriguingly, AnkX possesses a conserved FIC (filamentation induced by cyclic AMP) domain which was shown previously to harbor an AMPylation activity (Yarbrough et al., 2009). Next, the authors took advantage of high-resolution MS and obtained a highly accurate mass of the unknown moiety that was attached to Rab1. Through a thorough search of human metabolite database, a candidate molecule was suggested to be phosphocholine. Finally multi-stage MS (MS/MS/MS) analysis unambiguously confirmed the identity of this moiety. It is also noteworthy that MS data suggested <1% of total Rab1 proteins was actually modified during infection. This work to some extent best exemplified the power of high-sensitivity high-resolution MS technologies in PTM analyses.

Follow-up work on *Legionella*-mediated Rab1 modifications proved to be exciting as well. Two groups almost simultaneously reported the reversible reaction of AMPylation that was mediated by another effector SidD (Neunuebel et al., 2011; Tan and Luo, 2011). Moreover, phosphocholination can also be reversed by the *Legionella* effector Lem3 (Tan et al., 2011). Collectively, these studies provided a paradigm of bacteria-regulated reversible modifications of host factors and illustrated remarkable virulence mechanisms that are evolved by pathogenic bacteria. Nevertheless, it’s important to note that characterization of these newly discovered PTMs also illuminated novel signaling mechanisms that could be shared by mammalian host cells as well. For example, FIC domains are conserved in bacteria and eukaryotes including humans (Worby et al., 2009). Thus far, as many as 3000 Fic proteins have been found and they are likely to play important roles in diverse signaling processes, though we just started to understand only a few of their mechanisms (Engel et al., 2012).

## Closing remarks

The interplay of the host-pathogen system is highly complex, and traditional reductionist approaches have proved to be very fruitful in our understanding of molecular mechanisms underlying bacterial pathogenesis. Nevertheless, systems-level proteomic analyses offer us exciting new opportunities to investigate the intrinsically delicate balance of host-pathogen interactions. In transition from extracellular environment to their mammalian host, bacterial pathogens have evolved complex adaptation mechanisms to promote their survival and multiplication. From another perspective, host cells also mount extensive defense strategies in order to kill invading pathogens or at least contain infection. All these important aspects in host-pathogen interactions will be ultimately reflected in proteomic differences. MS-based analyses will likely contribute substantially to the elucidation of fundamental mechanisms of bacterial pathogenesis.

Nevertheless, it is important to be reminded that current proteomic technologies are not without limitations or challenges. In particular, comprehensive coverage of the host proteome is still rather difficult at this stage. Indeed, both studies on host protein expression during infection only measured a small subset of the entire proteome. Though some latest work demonstrated the capabilities of detecting >8000 proteins in eukaryotic cell lines (Geiger et al., 2012; Weekes et al., 2014), these studies are either labor-intensive or requiring large amounts of proteins to begin with. In many cases, these requirements can be quite demanding or difficult to meet, especially in the context of host-pathogen interactions. Therefore, further technical breakthroughs in proteomic platforms are certainly desired in terms of MS throughput as well as better chromatographic separations. In addition to incomplete proteome coverage, additional challenges may arise from translation of large volume of data into useful biological hypotheses. Development of bioinformatics tools have eased this problem to some extent, yet in many cases data interpretation can still be rate-limiting steps.

With current proteomic capabilities, success might be achieved first with bacterial pathogens considering their compact proteome. A clear trend is moving towards characterization of bacteria in the host. If combined with genetics, proteomic examination of effector-deficient mutants during infection might shed new light on functional redundancy of many bacterial effectors. Regarding host proteome, it should be emphasized again that the utility of proteomic datasets depends very much on the proteome coverage. In the meantime we will have to rely on extensive fractionations or subcellular enrichment prior to MS analyses to cover as many proteins as possible. Lastly for PTM analyses, novel enrichment techniques are needed if we want to move beyond common phosphorylation, glycosylation or ubiquitination. In summary, we believe that MS-based proteomics (or combined with other “omics” strategies and classic biological assays) holds the promise for systematic examination of molecular mechanisms underlying bacterial infection and thus helps develop new anti-microbial treatments in the near future.

## Abbreviations

LC-MS/MS: liquid chromatography-tandem mass spectrometry
MS: mass spectrometry
PTMs: post-translational modifications
SPI-1 and SPI-2: *Salmonella* Pathogenicity Island 1 and 2
T3SS: type III secretion system

## References

[CR1] Adkins JN, Mottaz HM, Norbeck AD, Gustin JK, Rue J, Clauss TRW, Purvine SO, Rodland KD, Heffron F, Smith RD (2006). Analysis of the *Salmonella* Typhimurium proteome through environmental response toward infectious conditions. Mol Cell Proteomics.

[CR2] Albrethsen J, Agner J, Piersma SR, Hojrup P, Pham TV, Weldingh K, Jimenez CR, Andersen P, Rosenkrands I (2013). Proteomic profiling of *Mycobacterium tuberculosis* identifies nutrient-starvation-responsive toxin-antitoxin systems. Mol Cell Proteomics.

[CR3] Ansong C, Yoon H, Norbeck AD, Gustin JK, McDermott JE, Mottaz HM, Rue J, Adkins JN, Heffron F, Smith RD (2008). Proteomics analysis of the causative agent of typhoid fever. J Proteome Res.

[CR4] Ansong C, Yoon H, Porwollik S, Mottaz-Brewer H, Petritis BO, Jaitly N, Adkins JN, McClelland M, Heffron F, Smith RD (2009). Global systems-level analysis of *Hfq* and *SmpB* deletion mutants in *Salmonella*: implications for virulence and global protein translation. PLoS One.

[CR5] Becker D, Selbach M, Rollenhagen C, Ballmaier M, Meyer TF, Mann M, Bumann D (2006). Robust *Salmonella* metabolism limits possibilities for new antimicrobials. Nature.

[CR6] Brown RN, Sanford JA, Park JH, Deatherage BL, Champion BL, Smith RD, Heffron F, Adkins JN (2012). A comprehensive subcellular proteomic survey of *Salmonella* grown under phagosome-mimicking versus standard laboratory conditions. Int J Proteomics.

[CR7] Bumann D (2009). System-level analysis of *Salmonella* metabolism during infection. Curr Opin Microbiol.

[CR8] Cash P (2011). Investigating pathogen biology at the level of the proteome. Proteomics.

[CR9] Cossart P, Sansonetti PJ (2004). Bacterial invasion: The paradigms of enteroinvasive pathogens. Science.

[CR10] Cravatt BF, Simon GM, Yates JR (2007). The biological impact of mass-spectrometry-based proteomics. Nature.

[CR11] Cui J, Yao Q, Li S, Ding X, Lu Q, Mao H, Liu L, Zheng N, Chen S, Shao F (2010). Glutamine deamidation and dysfunction of ubiquitin/NEDD8 induced by a bacterial effector family. Science.

[CR12] Curreem SO, Watt RM, Lau SK, Woo PC (2012). Two-dimensional gel electrophoresis in bacterial proteomics. Protein Cell.

[CR13] Di Pasqua R, Mamone G, Ferranti P, Ercolini D, Mauriello G (2010). Changes in the proteome of *Salmonella enterica serovar Thompson* as stress adaptation to sublethal concentrations of thymol. Proteomics.

[CR14] Ding C, Jiang J, Wei J, Liu W, Zhang W, Liu M, Fu T, Lu T, Song L, Ying W (2013). A fast workflow for identification and quantification of proteomes. Mol Cell Proteomics.

[CR15] Engel P, Goepfert A, Stanger FV, Harms A, Schmidt A, Schirmer T, Dehio C (2012). Adenylylation control by intra- or intermolecular active-site obstruction in Fic proteins. Nature.

[CR16] Fernandez-Arenas E, Cabezon V, Bermejo C, Arroyo J, Nombela C, Diez-Orejas R, Gil C (2007). Intergrated proteomics and genomics strategies bring new insight into *Candida albicans* response upon macrophage interaction. Mol Cell Proteomics.

[CR17] Fields BS, Benson RF, Besser RE (2002). *Legionella* and Legionnaires’ disease: 25 years of investigation. Clin Microbiol Rev.

[CR18] Galan JE, Wolf-Watz H (2006). Protein delivery into eukaryotic cells by type III secretion machines. Nature.

[CR19] Geiger T, Wehner A, Schaab C, Cox J, Mann M (2012). Comparative proteomic analysis of eleven common cell lines reveals ubiquitous but varying expression of most proteins. Mol Cell Proteomics.

[CR20] Gygi SP, Rist B, Gerber SA, Turecek F, Gelb MH, Aebersold R (1999). Quantitative analysis of complex protein mixtures using isotope-coded affinity tags. Nat Biotechol.

[CR21] Haraga A, Ohlson MB, Miller SI (2008). *Salmonellae* interplay with host cells. Nat Rev Microbiol.

[CR22] Hardwidge PR, Rodriguez-Escudero I, Goode D, Donohoe S, Eng J, Goodlett DR, Aebersold R, Finlay BB (2004). Proteomic analysis of the intestinal epithelial cell response to enteropathogenic *Escherichia coli*. J Biol Chem.

[CR23] Hartlova A, Krocova Z, Cerveny L, Stulik J (2011). A proteomic view of the host-pathogen interaction: the host perspective. Proteomics.

[CR24] Hoffmann C, Finsel I, Otto A, Pfaffinger G, Rothmeier E, Hecker M, Becher D, Hilbi H (2014). Functional analysis of novel Rab GTPase identified in the proteome of purified *Legionella*-containing vacuoles from macrophages. Cell Microbiol.

[CR25] Imami K, Bhavsar AP, Yu H, Brown NF, Rogers LD, Finlay BB, Foster LJ (2013). Global impact of *Salmonella* Pathogenicity Island 2-secreted effectors on the host phosphoproteome. Mol Cell Proteomics.

[CR26] Ingmundson A, Delprato A, Lambright DG, Roy CR (2007). *Legionella pneumophila* proteins that regulate Rab1 membrane cycling. Nature.

[CR27] Jafari M, Primo V, Smejkal GB, Moskovets EV, Kuo WP, Ivanov AR (2012). Comparison of in-gel protein separation techniques commonly used for fractionation in mass spectrometry-based proteomic profiling. Electrophoresis.

[CR28] Jenner RG, Young RA (2005). Insights into host responses against pathogens from transcriptional profiling. Nat Rev Microbiol.

[CR29] Kaper JB, Nataro JP, Mobley HLT (2004). Pathogenic *Escherichia coli*. Nat Rev Microbiol.

[CR30] Khabbaz RF, Moseley RR, Steiner RJ, Levitt AM, Bell BP (2014). Challenges of infectious diseases in the USA. Lancet.

[CR31] Kim K, Yang E, Vu GP, Gong H, Su J, Liu F, Lu S (2010). Mass spectrometry-based quantitative proteomic analysis of *Salmonella enterica* serovar enteritidis protein expression upon exposure to hydrogen peroxide. BMC Microbiol.

[CR32] Kotloff KL, Winichoff JP, Ivanoff B, Clemens JD, Swerdlow DL, Sansonetti PJ, Adak GK, Levine MM (1999). Global burden of *Shigella* infections: implications for vaccine development and implementation of control strategies. Bull World Health Organ.

[CR33] Kuntumalla S, Zhang Q, Braisted JC, Fleischmann RD, Peterson SN, Donohue-Rolfe A, Tzipori S, Pieper R (2011). *In vivo* versus *in vitro* protein abundance analysis of *Shigella dysenteriae* type 1 reveals changes in the expression of proteins involved in virulence, stress and energy metabolism. BMC Microbiol.

[CR34] Li Q (2011). Phagosome proteomics: a powerful tool to assess bacteria-mediated immunomodulation. Bioeng Bugs.

[CR35] Li H, Xu H, Zhou Y, Zhang J, Long C, Li S, Chen S, Zhou J, Shao F (2007). The phosphothreonine lyase activity of a bacterial type III effector family. Science.

[CR36] Li S, Zhang L, Yao Q, Li L, Dong N, Rong J, Gao W, Ding X, Sun L, Chen X (2013). Pathogen blocks host death receptor signalling by arginine GlcNAcylation of death domains. Nature.

[CR37] Liu X, Gao B, Novik V, Galan JE (2012). Quantitative proteomics of intracellular *Campylobacter jejuni* reveals metabolic reprogramming. PLoS Pathog.

[CR38] Machner MP, Isberg RR (2007). A bifunctional bacterial protein links GDI displacement to Rab1 activation. Science.

[CR39] Mukherjee S, Liu X, Arasaki K, McDonough J, Galan JE, Roy CR (2011). Modulation of Rab GTPase function by a protein phosphocholine transferase. Nature.

[CR40] Muller MP, Peters H, Bluemer J, Blankenfeldt W, Goody RS, Itzen A (2010). The *Legionella* effector protein DrrA AMPylates the membrane traffic regulator Rab1b. Science.

[CR41] Murata T, Delprato A, Ingmundson A, Toomre DK, Lambright DG, Roy CR (2006). The *Legionella pneumophila* effector protein DrrA is a Rab1 guanine nucleotide-exchange factor. Nat Cell Biol.

[CR42] Neunuebel MR, Chen Y, Gasper AH, Backlund PS, Yergey A, Machner MP (2011). De-AMPylation of the small GTPase Rab1 by the pathogen *Legionella pneumophila*. Science.

[CR43] Ohl ME, Miller SI (2001). *Salmonella*: a model for bacterial pathogenesis. Annu Rev Med.

[CR44] Paape D, Lippuner C, Schmid M, Ackermann R, Barrios-Llerena ME, Zimny-Arndt U, Brinkmann V, Arndt B, Pleissner KP, Jungblut PR (2008). Transgenic, fluorescent *Leishmania mexicana* allow direct analysis of the proteome of intracellular amastigotes. Mol Cell Proteomics.

[CR45] Pieper R, Zhang Q, Parmar PP, Huang ST, Clark DJ, Alami H, Donohue-Rolfe A, Fleischmann RD, Peterson SN, Tzipori S (2009). The *Shigella dysenteriae* serotype 1 proteome, profiled in the host intestinal environment, reveals major metabolic modifications and increased expression of invasive proteins. Proteomics.

[CR46] Pieper R, Fisher CR, Suh MJ, Huang ST, Parmar P, Payne SM (2013). Analysis of the proteome of intracellular *Shigella flexneri* reveals pathways important for intracellular growth. Infect Immun.

[CR47] Rabilloud T, Chevallet M, Luche S, Lelong C (2010). Two-dimensional gel electrophoresis in proteomics: past, present and future. J Proteomics.

[CR48] Rogers LD, Brown NF, Fang Y, Pelech S, Foster LJ (2011). Phosphoproteomic Analysis of *Salmonella*-infected cells identifies key kinase regulators and SopB-dependent host phosphorylation events. Sci Signal.

[CR49] Salomon D, Orth K (2013). What pathogens have taught us about posttranslational modifications. Cell Host Microbe.

[CR50] Schmidt F, Volker U (2011). Proteome analysis of host-pathogen interactions: investigation of pathogen responses to the host cell environment. Proteomics.

[CR51] Schmutz C, Ahrne E, Kasper CA, Tschon T, Sorg I, Dreier RF, Schmidt A, Arrieumerlou C (2013). Systems-level overview of host protein phosphorylation during *Shigella flexneri* infection revealed by phosphoproteomics. Mol Cell Proteomics.

[CR52] Schoebel S, Oesterlin LK, Blankenfeldt W, Goody RS, Itzen A (2009). RabGDI displacement by DrrA from *Legionella* is a consequence of its guanine nucleotide exchange activity. Mol Cell.

[CR53] Sengupta N, Alam SI (2011). In vivo studies of *Clostridium perfringens* in mouse gas gangrene model. Curr Microbiol.

[CR54] Sherwood RK, Roy CR (2013). A Rab-centric perspective of bacterial pathogen-occupied vacuoles. Cell Host Microbe.

[CR55] Shi L, Adkins JN, Coleman JR, Schepmoes AA, Dohnkova A, Mottaz HM, Norbeck AD, Purvine SO, Manes NP, Smallwood HS (2006). Proteomic analysis of *Salmonella enterica* serovar Typhimurium isolated from RAW 264.7 macrophages - Identification of a novel protein that contributes to the replication of serovar Typhimurium inside macrophages. J Biol Chem.

[CR56] Shi L, Ansong C, Smallwood H, Rommereim L, McDermott JE, Brewer HM, Norbeck AD, Taylor RC, Gustin JK, Heffron F (2009). Proteome of *Salmonella enterica* serotype Typhimurium grown in a low Mg/pH medium. J Proteomics Bioinform.

[CR57] Shi L, Chowdhury SM, Smallwood HS, Yoon H, Mottaz-Brewer HM, Norbeck AD, McDermott JE, Clauss TRW, Heffron F, Smith RD (2009). Proteomic investigation of the time course responses of RAW 264.7 macrophages to infection with *Salmonella enterica*. Infect Immun.

[CR58] Sonck KAJ, Kint G, Schoofs G, Vander Wauven C, Vanderleyden J, De Keersmaecker SCJ (2009). The proteome of *Salmonella* Typhimurium grown under in vivo-mimicking conditions. Proteomics.

[CR59] Stancik LM, Stancik DM, Schmidt B, Barnhart DM, Yoncheva YN, Slonczewski JL (2002). pH-dependent expression of periplasmic proteins and amino acid catabolism in *Escherichia coli*. J Bacteriol.

[CR60] Suh MJ, Kuntumalla S, Yu Y, Pieper R (2014). Proteomes of pathogenic *Escherichia coli/Shigella* group surveyed in their host environments. Expert Rev Proteomics.

[CR61] Tan Y, Luo Z (2011). *Legionella pneumophila* SidD is a deAMPylase that modifies Rab1. Nature.

[CR62] Tan Y, Arnold RJ, Luo Z (2011). *Legionella pneumophila* regulates the small GTPase Rab1 activity by reversible phosphorylcholination. Proc Natl Acad Sci USA.

[CR63] Thingholm TE, Jensen ON, Larsen MR (2009). Analytical strategies for phosphoproteomics. Proteomics.

[CR64] Twine SM, Mykytczuk NCS, Petit MD, Shen H, Sjostedt A, Conlan JW, Kelly JF (2006). In vivo proteomic analysis of the intracellular bacterial pathogen, *Francisella tularensis*, isolated from mouse spleen. Biochem Biophys Res Commun.

[CR65] Urwyler S, Nyfeler Y, Ragaz C, Lee H, Mueller LN, Aeversold R, Hilbi H (2009). Proteome analysis of *Legiobella* vacuoles purified by magnetic immunoseparation reveals secretory and endosomal GTPases. Traffic.

[CR66] Walduck A, Rudel T, Meyer TF (2004). Proteomic and gene profiling approaches to study host responses to bacterial infection. Curr Opin Microbiol.

[CR67] Weber A, Kogl SA, Jung K (2006). Time-dependent proteome alterations under osmotic stress during aerobic and anaerobic growth in *Escherichia coli*. J Bacteriol.

[CR68] Weekes MP, Tomasec P, Huttlin EL, Fielding CA, Nusinow D, Stanton RJ, Wang EC, Aicheler R, Murrell I, Wilkinson GW (2014). Quantitative temporal viromics: an approach to investigate host-pathogen interaction. Cell.

[CR69] Worby CA, Mattoo S, Kruger RP, Corbeil LB, Koller A, Mendez JC, Zekarias B, Lazar C, Dixon JE (2009). The fic domain: regulation of cell signaling by adenylylation. Mol Cell.

[CR70] Yarbrough ML, Li Y, Kinch LN, Grishin NV, Ball HL, Orth K (2009). AMPylation of Rho GTPases by Vibrio VopS disrupts effector binding and downstream signaling. Science.

[CR71] Yohannes E, Barnhart DM, Slonczewski JL (2004). pH-dependent catabolic protein expression during anaerobic growth of *Escherichia coli* K-12. J Bacteriol.

[CR72] Yu J, Guo L (2011). Quantitative proteomic analysis of *Salmonella enterica* serovar Typhimurium under PhoP/PhoQ activation conditions. J Proteome Res.

[CR73] Zhang CG, Chromy BA, McCutchen-Maloney SL (2005). Host-pathogen interactions: a proteomic view. Expert Rev Proteomics.

[CR74] Zhang L, Ding X, Cui J, Xu H, Chen J, Gong Y, Hu L, Zhou Y, Ge J, Lu Q (2011). Cysteine methylation disrupts ubiquitin-chain sensing in NF-κB activation. Nature.

[CR75] Zhu L, Zhao G, Stein R, Zheng X, Hu W, Shang N, Bu X, Liu X, Wang J, Feng E (2010). The proteome of *Shigella flexneri* 2a 2457T grown at 30 and 37°. Mol Cell Proteomics.

